# KNOWLEDGE OF END-OF-LIFE CARE, LIFE-SUSTAINING TREATMENT, AND ADVANCE DIRECTIVES IN NURSING STUDENTS

**DOI:** 10.1093/geroni/igz038.2663

**Published:** 2019-11-08

**Authors:** Jooyoung Cheon

**Affiliations:** Sungshin Women’s University, Seoul, Korea, Republic of

## Abstract

Knowledge of end-of-life (EOL) care and being able to make the appropriate decisions for patients who are dying is important for nursing students, who will soon play a critical role in EOL decision-making among patients and their caregivers. Accordingly, the purpose of this study was to examine the level of knowledge of EOL care, life-sustaining treatment, and advance directives among nursing students in South Korea. This cross-sectional descriptive study was conducted from December 2017 to February 2018. Data were collected from 220 undergraduate nursing students and analyzed using descriptive statistics, t-tests, one-way ANOVA, and a post hoc test with the SPSS 19.0 program. The score for knowledge of EOL care was 7.8 out of 11 points, for knowledge of life-sustaining treatment was 4.6 out of 6 points, and for knowledge of advance directives was 7.0 out of 9 points. There were significant differences in knowledge of EOL care scores by year of study, experiences in clinical practicum education, and experiences of caring for dying patients. Knowledge of life-sustaining treatment significantly differed by year of study, experiences in clinical practicum education, experiences of caring for and observing dying patients during clinical practicum education, and perceived self-rated health. There were significant differences in knowledge of advance directive scores by year of study, satisfaction with nursing major, experiences in clinical practicum education, and experiences of caring for and observing dying patients during clinical practicum education. Further studies should develop educational intervention programs that improve knowledge of EOL care, life-sustaining treatment, and advance directives.

